# XML-BSPM: an XML format for storing Body Surface Potential Map recordings

**DOI:** 10.1186/1472-6947-10-28

**Published:** 2010-05-14

**Authors:** Raymond R Bond, Dewar D Finlay, Chris D Nugent, George Moore

**Affiliations:** 1 Computer Science Research Institute, School of Computing and Mathematics, University of Ulster, Shore Road, Newtownabbey, Co. Antrim, BT37 0QB, UK

## Abstract

**Background:**

The Body Surface Potential Map (BSPM) is an electrocardiographic method, for recording and displaying the electrical activity of the heart, from a spatial perspective. The BSPM has been deemed more accurate for assessing certain cardiac pathologies when compared to the 12-lead ECG. Nevertheless, the 12-lead ECG remains the most popular ECG acquisition method for non-invasively assessing the electrical activity of the heart. Although data from the 12-lead ECG can be stored and shared using open formats such as SCP-ECG, no open formats currently exist for storing and sharing the BSPM. As a result, an innovative format for storing BSPM datasets has been developed within this study.

**Methods:**

The XML vocabulary was chosen for implementation, as opposed to binary for the purpose of human readability. There are currently no standards to dictate the number of electrodes and electrode positions for recording a BSPM. In fact, there are at least 11 different BSPM electrode configurations in use today. Therefore, in order to support these BSPM variants, the XML-BSPM format was made versatile. Hence, the format supports the storage of custom torso diagrams using SVG graphics. This diagram can then be used in a 2D coordinate system for retaining electrode positions.

**Results:**

This XML-BSPM format has been successfully used to store the Kornreich-117 BSPM dataset and the Lux-192 BSPM dataset. The resulting file sizes were in the region of 277 kilobytes for each BSPM recording and can be deemed suitable for example, for use with any telemonitoring application. Moreover, there is potential for file sizes to be further reduced using basic compression algorithms, i.e. the deflate algorithm. Finally, these BSPM files have been parsed and visualised within a convenient time period using a web based BSPM viewer.

**Conclusions:**

This format, if widely adopted could promote BSPM interoperability, knowledge sharing and data mining. This work could also be used to provide conceptual solutions and inspire existing formats such as DICOM, SCP-ECG and aECG to support the storage of BSPMs. In summary, this research provides initial ground work for creating a complete BSPM management system.

## Background

### The Body Surface Potential Map

The Body Surface Potential Map (BSPM) is a specialised electrocardiographic method, for recording and displaying the electrical activity of the heart, from a spatial perspective. The BSPM has been deemed more accurate for diagnosing certain cardiac pathologies, when compared to the 12-lead electrocardiogram (ECG) [1,2]. The major advantage of the BSPM is its ability to display electrical information in the spatial domain. This is achieved by placing a large number of electrodes (32-213) around the human torso, whereas the 12-lead ECG utilizes six thoracic electrodes which are subject to a limited anatomical area, namely the precordium [3].

### Importance of interoperability

Interoperability is an important area of research, since it promotes the intercommunication of clinical documents between heterogeneous hospital information systems [4]. The predominant driver in promoting interoperability has been the development of open formats for storing clinical information [5]. These open formats can be easily integrated into the Electronic Patient Health Record (EPHR). Moreover, according to Fischer *et al*. [6], cardiological information is progressively being introduced into the EPHR. This is just one reason why a BSPM format should be created. Although the BSPM has been clinically proven to be more accurate for diagnosing cardiac patients, no work has been undertaken to improve the interoperability of BSPM data. Scientists currently store BSPM datasets in a number of custom formats which include propriety data files (e.g. MATLAB, Map3D) and format specific files (formatted Comma Separated Values).

### ECG formats

A plethora of open formats have been created for storing and transmitting the ECG. These formats have been based around more familiar recording methods such as the 12-lead ECG. The three major industrial ECG formats exist; the Digital Imaging and Communication in Medicine ECG (DICOM-ECG) format; Health Level 7 Annotated ECG (HL7/aECG) and the Standardised Communication Protocol ECG (SCP-ECG) format. Other influential ECG formats include ecgML [7], XML-ECG [8], MFER [9], to name but a few. The following Sections provide a more detailed overview of each of the aforementioned three major formats.

### DICOM-ECG

The DICOM standard, formally known as ACR-NEMA was created by the National Electrical Manufactures Association (NEMA) in 1985 [10,11]. ACR-NEMA evolved as DICOM version 3 in 1993, and became a European standard in 1995. The DICOM format originally stored radiographic raster images, from diagnostic devices such as the X-RAY [12]. DICOM now endeavours to support all diagnostic modalities including the ECG. As a result, DICOM waveform supplement 30 was introduced in the year 2000. This extension enables the storage of raw waveform datasets i.e. blood pressure, audio and the ECG. Within the ECG community, DICOM supplement 30 is also called DICOM-ECG.

### HL7

The Annotated ECG (aECG) format was created in partnership between the Health Level 7 and the US Food and Drug Administration (FDA) in 2001 [13]. It was then accepted as a standard by the American National Standards Institute (ANSI) in 2004. The FDA where collecting a large number of ECGs, submitted by pharmaceutical companies for clinical trials. These ECGs where submitted in various formats, some of which where hard copies that had to be scanned for electronic storage. The aECG format was therefore created to improve the administration tasks of managing such a complicated process. This was the first ECG format based on the eXtensible Markup Language (XML).

### SCP-ECG

The SCP-ECG format is a compressed binary based format that takes advantage of Huffman encoding. In 2002, SCP-ECG became a promotion of the European funded OpenECG consortium [5]. The OpenECG network is a body of people dedicated to the interoperability in digital electrocardiography. According to Chronaki *et al., *they have at least 464 members [14]. In 2005, the SCP-ECG format became the official European standard for the storage and transmission of ECGs [5,14].

All of the aforementioned ECG formats are non proprietary and are therefore capable of achieving interoperability, whereas many ECG Management Systems (EMS) integrate closed proprietary formats into the EPHR. These closed formats include Unipro, Sifor and MDW [15].

It has been shown that none of the aforementioned ECG formats support the storage and transmission of BSPMs. This is likely to be caused by the fact that current ECG formats specialise in catering for popular ECG acquisition methods, such as the 12-lead ECG. It may also be difficult to create a format that supports both the 12-lead ECG and the BSPM, since the requirements for storing either are considerably different. This is illustrated in Table 1.

**Table 1:** Storage requirements

	12-lead ECG	BSPM
***Electrodes***	10	32-213

***Bipolar leads***	3	0

***Unipolar leads***	9	32-213 *All unipolar, each electrode has an associated unipolar lead.

***Electrode positions***	Standardized.	Non-standardized *Range of different layouts.

***Calculated leads***	Standard calculations for generating limb leads aVF, aVL, aVR and III.	Non standardized * Range of limited lead sets, for example the Lux-32 layout expands to Lux-192 using coefficients.

***Data***	Usually a 10 second recording.	No set recording time. Some BSPMs store single beats.

***Formatting requirements***	Requires a strict standard format since the 12-lead ECG is well standardized.	Requires a versatile format to support a range of electrode layouts.

***Display***	12 waveforms are usually rendered onto formatted graph paper, i.e. "3 × 4 + 1".	No standard representation exists. Can be displayed as a series of scalar traces or as a contour map. * Electrode positions must be known to generate a contour map.

***Diagnostic criteria***	Diagnostic criteria have been well defined.	Very little diagnostic criteria exist.

***Accessibility and cost of equipment***	Very accessible within the healthcare industry and relatively inexpensive.	Not all hospitals have a BSPM recording and visualisation system. Equipment can be expensive and hard to get.

The difference in configuration and storage requirements between the 12-lead ECG and the BSPM.

**Figure 1:**
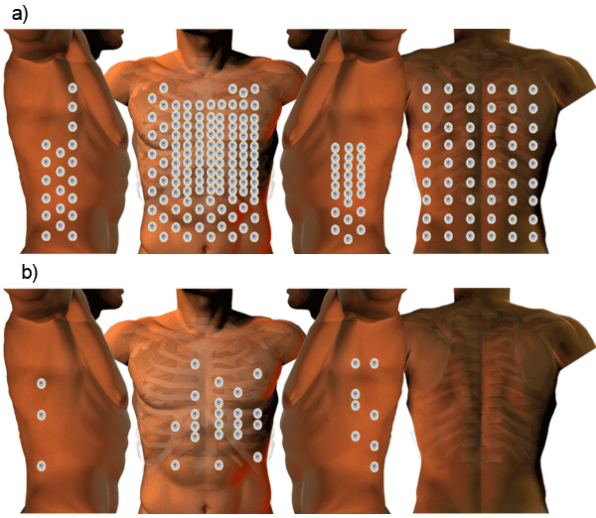
**BSPM electrode configuration**. This diagram illustrates the diversity in BSPM electrode configuration. a) This represents the anatomical locations of the 219 electrodes employed by the Parma lead set. b) This represents the anatomical locations of the 32 electrodes employed by the Lux Anterior lead set.

One of the major issues is that, the methods of recording, processing and displaying BSPMs have not yet been standardised [16]. A BSPM can be recorded using an arbitrary number of electrodes placed at customised anatomical locations. According to Hoekema *et al*. there are at least 11 international electrode layouts that are in use today, some of which use anywhere between 32 (Lux-32 Anterior BSPM) and 219 (Parma-219 BSPM) electrodes as illustrated in Figure 1[16]. In addition, since the number electrodes in BSPM acquisition has not been dictated by a standards consortium, researchers may have developed their own custom electrode layouts for BSPM acquisition.

In contrast to the BSPM, the 12-lead ECG has been standardised since 1938 [17]. The 12-lead ECG utilizes 10 electrodes, which are positioned at well defined anatomical locations [18]. These include six chest electrodes which are placed at specific well defined landmarks on the precordium. It is these specifics that have made it easy to develop storage formats for the 12-lead ECG. Conversely, it is the lack of specifics, hence versatility in BSPM acquisition that leaves the community with the huge challenge of defining a format that supports all BSPM variations. Out of the existing formats, SCP-ECG supports up to 255 leads. These are, however, predefined leads, i.e. right sided chest leads [19]. The format is therefore not versatile enough to support BSPMs. Likewise, a simple change to the aECG specification would enable the storage of an arbitrary number of leads. Unfortunately, this would still leave the problem of storing the actual electrode positions. Given that the current ECG formats focus on storing 12-lead ECG data, such formats do not retain electrode positions because they are standardised and can be easily observed in clinical literature.

**Figure 2:**
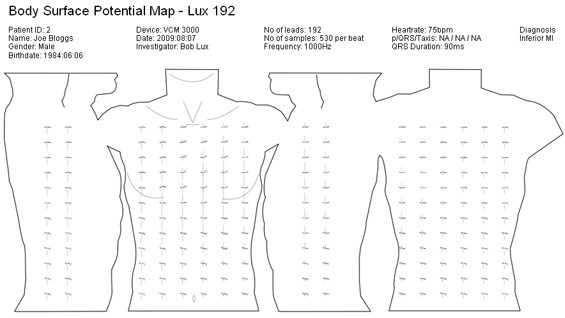
**Example BSPM**. An unrolled 2D torso diagram displaying 192 averaged beats.

BSPM datasets can be displayed as a contour map, a series of scalar traces or as a set of averaged beats. Waveforms are usually positioned over their associated electrode positions using a simple 2D unrolled torso diagram. This is illustrated in Figure 2. There is no standard torso diagram in place for displaying BSPMs. Researchers currently draw their own custom torso diagrams for displaying BSPM data. Therefore, the three main challenges in creating a BSPM format are:

1. Supporting custom torso diagrams.

2. Storing an arbitrary number of leads.

3. Storing the associated electrode positions for each lead.

From these three challenges, storing electrode positions is the most challenging and arguably the most important aspect of a BSPM format. Electrode positions are required for clinical reference and for visualising the BSPM data, i.e. contour plotting.

## Methods

There are a number of options when developing a solution to the BSPM storage problem. One option is to propose a BSPM extension to one of the current ECG formats such as SCP-ECG. Another option is to create a new BSPM format. The former option promotes the philosophy of supporting all ECG acquisition methods using a single format. This is similar to the DICOM philosophy, where the aim is to support all diagnostic modalities under one standard. The latter option involves the creation of a specialised format that will only store one ECG acquisition method, namely the BSPM.

Within our current work we have opted to adopt the strategy of creating a specialised format. This decision is partially attributed to the fact that there has been a development in growth in specialised ECG formats. These formats include ecgAware [20], which concentrates on storing ambulatory ECG data, and mECG [21] which concentrates on storing ECG data for mobile devices. Goncalves *et al. *created the ecgAware format because existing formats such as aECG and ecgML do not support ambulatory ECG monitoring, i.e. Holter monitoring. It is not obvious whether the advantages of general formats like DICOM outweigh the advantages of specific formats such as the ecgAware format. General formats are usually more complex, since they manage a large specification to support a range of modalities, whereas specific formats do not have this complication. Furthermore, since BSPMs are not as standardised as other ECG acquisition methods, a specialised BSPM format is more appropriate rather than integrating a complex BSPM extension into an existing format. If the proposed BSPM format is not industrialised, then this work, at least, provides the rationale for extending an existing format.

### Implementation Method

**Figure 3:**
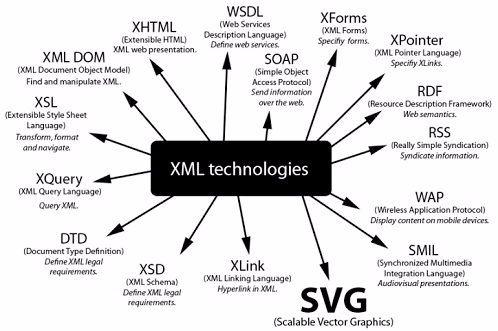
**XML technologies**. This diagram depicts a plethora of XML related technologies that can be combined with XML-BSPM.

XML and binary are the two predominant implementation methods for storing ECG data. SCP-ECG stores data using a binary based format, whereas the aECG format uses XML. Binary formats are usually smaller in terms of file size, however, disk space and transmission bandwidth requirements are not as much of a major issue as they once were and XML compression techniques do exist [22]. Although XML is more verbose, it does innately benefit from human readability, whereas a computer program is needed to read a binary based format such as SCP-ECG. Unlike binary, XML has the advantage of possibly exploiting a plethora of related XML technologies. These related XML technologies are illustrated in Figure 3. Regarding ECG formats, there has been a growth in XML based formats which may indicate the strength of XML [7,8,13]. Moreover, the power of XML within the healthcare industry has been well documented [23]. XML is also promoted by CDISC's Submission Data Model (SDM) [24].

**Figure 4:**
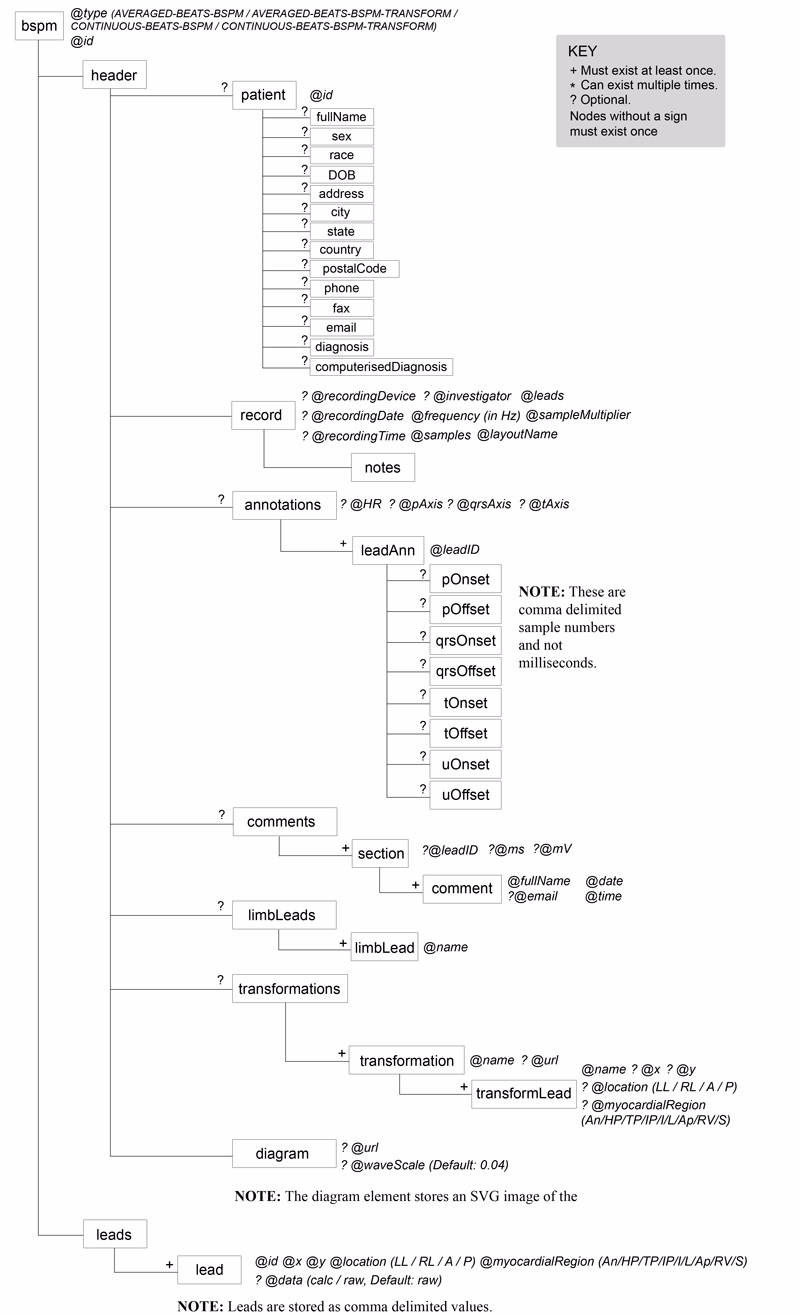
**BSPM XML tree structure**. Overview of the BSPM XML tree structure. The format is split into two main sections, the ***header***, which retains the meta-data and the leads section, which stores the actual ECG values.

As a result of the aforementioned rationale, an XML based format has been created within this study for storing BSPMs. Figure 4 represents the overall tree structure of this format.

### The root element

The root element ***bspm ***stores two required attributes called ***type ***and ***id***. The attribute ***type ***defines what kind of BSPM data the file is storing. It therefore, indicates to a human observer or a computer program the type of data it is to expect. This is important as there are different methods for recording BSPM data. The ***type ***attribute can have one of four values (***AVERAGED-BEATS-BSPM, AVERAGED-BEATS-BSPM-TRANSFORM, CONTINUOUS-BSPM ***or ***CONTINUOUS -BSPM-TRANSFORM***). The value ***AVERAGED-BEATS-BSPM ***is used when each ***lead ***element within the format stores one single beat as raw number values, whereas the value ***AVERAGED-BEATS-BSPM-TRANSFORM ***indicates that some leads are derived, in that they contain equations as opposed to raw number values. Supporting derived leads is important because there has been substantial research carried out within the ECG community that involves limited lead sets and BSPM transformations [25]. The value ***CONTINUOUS-BEATS-BSPM***, as the name suggests, is used when continuous data is stored for each lead. The value ***CONTINUOUS-BSPM-TRANSFORM ***is used to indicate the storage of multiple beats, some of which are stored in raw form and the derived leads are stored as mathematical equations. The attribute ***id ***is used to either uniquely identify a document or to associate the file with a database.

The ***bspm ***element has two sub elements called ***header ***and ***leads***. The ***header ***element stores the metadata. That is, information about the data, which includes patient demographics, record settings, annotations etc. The ***leads ***element stores the actual BSPM lead data. A full description of the root element can be seen in Table 2.

**Table 2:** Description of the root element (*bspm*)

Attributes			
**Name**	**Required**	**Data type**	**Description**

***type***	Required	AVERAGED-BEATS-BSPM/AVERAGED- BEATS -BSPM-TRANSFORM/CONTINUOUS-BSPM/CONTINUOUS - BSPM-TRANSFORM	This format can store four types of BSPMs.

***id***	Required	String	For associating the file with a database.

**Elements**			

**Name**	**Required**	**Data type**	**Description**

***header***	Required	See header table.	This element separates the data from metadata, presenting the format in a coherent fashion.

***leads***	Required	See leads table.	This element groups the entire lead data. It has been named leads as opposed to channels because all BSPM leads are unipolar.

### The header element

The ***header ***element does not store any attributes or data of its own. Instead, it is used as a wrapper element to group all of the metadata as sub elements. This separates the metadata from the actual lead data and improves readability of the XML document. The ***header ***element can have up to seven sub elements (***patient***, ***record***, ***annotations***, ***comments***, ***limbLeads***, ***transformations ***and ***diagram***) and is described in Table 3.

**Table 3:** Description of the header element

Elements			
**Name**	**Required**	**Data type**	**Description**

***patient***	Required	See patient table.	Patient demographics and diagnosis.

***record***	Required	See record table.	Information about the recording settings, i.e. device, time, and recording physician etc.

***annotations***	Required	See annotations table.	This is a wrapper element for storing beat markers, i.e. p onset.

***comments***	Optional	See comments table.	This can be used for collaboration and discussion amongst clinicians.

***limbLeads***	Optional	See limbLeads table.	Some BSPM datasets retain the limb leads that were used to calculate the Wilson Central Terminal (WCT). These limb leads can be stored here and may prove useful in post processing.

***transformations***	Optional	See transformations table.	This is where equations can be stored to transform the BSPM into the 12-lead ECG or the VCG.

***diagram***	Required	CDATA, see diagram table for attributes.	This element stores an SVG diagram that represents an unrolled 2D torso.

The ***patient ***element is optional given that some BSPM datasets may be associated with a database, which may already retain the patient demographics. In this case, a duplicate would not be necessary. The ***patient ***element retains one attribute called ***id***, which can be used to uniquely identify a patient. Moreover, the ***patient ***element has a number of sub elements (***fullname***, ***sex***, ***DOB***, ***address ***etc.) as listed in Figure 4. These elements provide the basic information that is needed to fully identify a patient. A full description of the ***patient ***element is depicted in Table 4.

**Table 4:** Description of the patient element

Attributes			
**Name**	**Required**	**Data type**	**Description**

***id***	Required	String	This is used to uniquely identify a patient, e.g. 0000001.

**Elements**			

**Name**	**Required**	**Data type**	**Description**

***fullName***	Optional	String	Name of the patient, e.g. John Smith.

***sex***	Optional	male/female/unspecified/unknown	E.g. male.

***DOB***	Optional	Date YYYY-MM-DD	E.g. 1984-06-24.

***address***	Optional	String	E.g. 47 University street

***city***	Optional	String	E.g. Belfast

***state***	Optional	String	E.g. Antrim


***country***	Optional	String	E.g. Northern Ireland

***postalCode***(ZipCode)	Optional	String	This element can store either a postcode or a Zipcode, e.g. BT37 0QB.

***phone***	Optional	String	The data type is string to support commas and hyphens, e.g. 08 700 400 700.

***fax***	Optional	String	E.g. 08 700 400 700.

***email***	Optional	String	E.g. johnsmith@myemailhost.com

***diagnosis***	Optional	String	This element is for storing the patient's diagnosis, as perceived by a clinician, e.g. Acute Myocardial Infarction.

***computerisedDiagnosis***	Optional	String	This element is used to record the predicted diagnosis made by a computerised classification algorithm, e.g. Left Bundle Branch Block.

**Figure 5:**
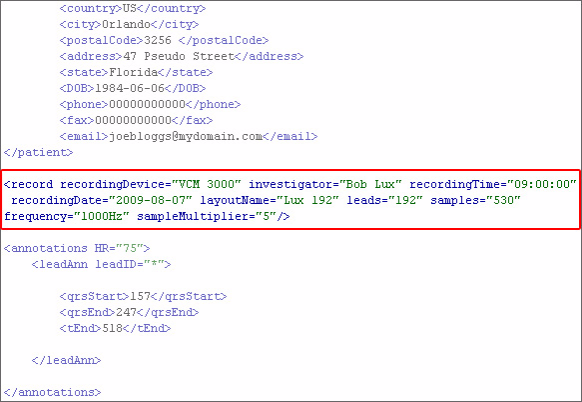
**record element**. An XML excerpt of the record element, illustrating the readability of its attributes.

The ***record ***element is required as it stores important information which is necessary for the visualisation of the data. For example, the ***frequency ***of a BSPM recording must be known, in order to draw the actual waveforms. Most of the information, within the ***record ***element is stored as attributes, as opposed to elements, as it improves the readability of the XML document and reduces the file size. Figure 5 is an XML excerpt of the ***record ***element and illustrates how condensed and readable the ***record ***element can be. The ***record ***element can have nine attributes, five of which are optional (***recordingDevice, recordingTime, recordingDate, investigator, mVSteps***) and four that are required (***layoutName, leads, samples, frequency***). The ***recordingDevice ***attribute stores the name of the device that was used for recording the BSPM. This attribute is not required, but such information may be helpful when comparing different recording devices. The attributes ***recordingTime ***and ***recordingDate ***store the time and date of when the actual BSPM recording took place. These are also optional given that this information may have been disregarded. However, the time and date of a diagnostic procedure is important in most clinical scenarios. The ***investigator ***attribute stores the full name of the clinician who carried out the procedure. This is an optional attribute due to the fact that procedures cannot be attributed to only one investigator. The ***layoutName ***attribute is required as there are many electrode layouts that can be used in a BSPM acquisition. Therefore, it is important to define which electrode layout was used. The ***leads ***attribute is required because it stores the number of leads that have been stored within the document. This number excludes any limb leads that have been stored within the ***header ***element. The next three attributes (***samples, frequency and sampleMultiplier***) are important, since their values assist in rendering the actual waveforms. The ***samples ***attribute stores the number of sample values that have been stored in each lead. The ***frequency ***attribute stores the value of the sample frequency in Hz. Finally, the ***sampleMultiplier ***attribute stores a number which is used for mathematically multiplying and hence magnifying each of the sample values. This attribute has been added to support legacy datasets where sample values need to be multiplied by such a number in order to retrieve the actual values. This value is used to improve the resolution of the waveforms. Although this attribute is optional, if it is not present, the value defaults to 1. The record element can have one sub element, called ***notes***. This element gives the investigator or the technician the option to insert a detailed description of the recording procedure. A full description of the ***record ***element can be seen in Table 5.

**Table 5:** Description of the record element

Attributes			
**Name**	**Required**	**Data type**	**Description**

***recordingDevice***	Optional	String	The name of the device used to record the BSPM, e.g. VCM-3000.

***recordingTime***	Optional	Time:HH:MM:SS:SSS	The time of day the recording took place, e.g. 12:50:00:000.

***recordingDate***	Optional	Date YYYY-MM-DD	The date the recording took place, e.g. 2009-08-28.

***investigator***	Optional	String	The name of the recording clinician, e.g. Fred Kornreich.

***layoutName***	Required	String	The name of BSPM configuration method, e.g. Lux-192.

***leads***	Required	Integer	This records the number of leads that have been stored within the leads element, e.g. 192.

***samples***	Required	Integer	This stores the number of sample values that have been stored for each lead, e.g.600.

***frequency***	Required	String	Number of samples per second recorded in Hertz, e.g. "1000 Hz" can be interpreted as 1000 samples per second.

***sampleMultiplier***	Optional	Float DEFAULT: 1	This is the number each sample value must be multiplied by, in order to get the actual value. If this attribute does not exist, the value defaults to 1.

**Elements**			

**Name**	**Required**	**Data type**	**Description**

***notes***	Optional	String	This allows the investigator to include notes that best describe the recording procedure.

The ***annotations ***element is optional, but in many cases can be important for post processing and clinical analysis. For example, an ST40 isopotential map is commonly used to assist in diagnosing ischemic disorders. A map such as this cannot be easily generated if the relevant annotations have not been defined within the format. The ***annotations ***element can store four optional attributes. The ***HR ***attribute is an abbreviation for heart rate. The heart rate can be stored within the format given that a lot of BSPM datasets store one beat and at least two beats are required to calculate the heart rate using the R-R interval. Therefore, this attribute allows the clinician to calculate the heart rate and store it if only single beats are retained. The other attributes ***pAxis***, ***qrsAxis ***and ***tAxis ***store the cardiac axis in degrees. We have facilitated the storage of cardiac axis as it cannot be easily calculated from the non standard leads that often make up a BSPM. The ***annotations ***element has one sub element, namely ***leadAnn***. The ***leadAnn ***element stands for lead annotation. This element must exist at least once, but can also exist multiple times. A description of the ***annotations ***element can be seen in Table 6.

**Table 6:** Description of the annotations element

Attributes			
**Name**	**Required**	**Data type**	**Description**

***HR***	Optional	Integer	The Heart Rate can be stored, because a patient's heart rate can not be calculated from a single beat, e.g. 70 bpm.

***pAxis***	Optional	Integer	In degrees, e.g. 40°.

***qrsAxis***	Optional	Integer	In degrees, e.g. 70°.

***tAxis***	Optional	Integer	In degrees, e.g. 50°.

**Elements**			

**Name**	**Required**	**Data type**	**Description**

***leadAnn***	Required	See leadAnn table.	leadAnn stands for lead annotation.

The ***leadAnn ***element acts as a wrapper tag that groups all of the beat markers, i.e. ***pOnset***, ***pOffset ***etc. The ***leadAnn ***element has one required attribute called ***leadID***. This ***leadID ***attribute stores a value that corresponds with the ***id ***attribute of the ***lead ***element. This means, each lead can have its own independent beat markers. However, most BSPM datasets use the same beat markers for all the leads. If this is the case, the value "*" can be ascribed to the ***leadID ***attribute. This value is commonly used in computer science to select all the items in concern. Therefore in this context, the value "*" would instruct a computer program to use the defined beat markers for all leads. The ***leadAnn ***element can have eight sub elements (***pOnset, pOffset, qrsOnset, qrsOffset ***etc). Each sub element stores sample values that define beat markers, respectively. For example, the ***pOnset ***element stores sample numbers that refer to the p wave onset locations. These eight sub elements will each store a single value if the BSPM being stored is a set of averaged single beats. On the contrast, multiple values are stored in a CSV format if the BSPM being stored is a set of multiple beats. A full description of the ***leadAnn ***element can be seen in Table 7.

**Table 7:** Description of the leadAnn element

Attributes			
**Name**	**Required**	**Data type**	**Description**

***leadID***	Required	String	This value corresponds to the ***id ***attribute stored within each ***lead ***element. This attribute is used to identify which lead the beat markers are referring to. The value * means that the beat markers refer to all the leads.

**Elements**			

**Name**	**Required**	**Data type**	**Description**

***pOnset***	Optional	String	Stores the onset of the P deflection. Multiple values are stored as CSV.

***pOffset***	Optional	String	Stores the offset of the P deflection. Multiple values are stored as CSV.

***qrsOnset***	Optional	String	Stores the onset of the QRS deflection. Multiple values are stored as CSV.

***qrsOffset***	Optional	String	Stores the offset of the QRS deflection. Multiple values are stored as CSV.

***tOnset***	Optional	String	Stores the onset of the T deflection. Multiple values are stored as CSV.

***tOffset***	Optional	String	Stores the offset of the T deflection. Multiple values are stored as CSV.

***uOnset***	Optional	String	Stores the onset of theU deflection. Multiple values are stored as CSV.

***uOffset***	Optional	String	Stores the offset of the U deflection. Multiple values are stored as CSV.

The ***comments ***element allows an observer or multiple observers to store textual information alongside the waveform data. This feature can be used for collaboration and discussion, since evaluating a BSPM is still a relatively new process and is not, as yet, a fine art. The ***comments ***element does not have any attributes, but it has one sub element called ***section***. This element can exist multiple times within the ***comments ***element. The ***section ***element is used to group comments that refer to the same section of the same lead. This way, comments can be formatted and presented similarly to online forums. The ***section ***element has three optional attributes. The ***leadID ***attribute makes reference to a particular ***lead ***within the ***leads ***element by storing the ***id ***of a ***lead***. If a comment is being referred to the whole BSPM, the "*" can be attributed to the ***leadID ***attribute. The attributes ***ms ***(milliseconds) and ***mV ***(microvolts) are also optional. These attributes specify which particular portion of a lead; the enclosed comment(s) are referring to. The ***section ***element has one sub element called ***comment***, which must exist at least once. This element stores the actual textual information inserted by a clinician. A full description of the ***section ***element can be viewed in Table 8.

**Table 8:** Description of the section element

Attributes			
**Name**	**Required**	**Data type**	**Description**

***leadID***	Optional	String	This attribute is used to specify which lead the sub comments refer to. If a comment refers to all leads, the value "*" can be used.

***ms***	Optional	Float	This ms (milliseconds) attribute specifies which part of the waveform the sub comments are referring to.

***mV***	Optional	Float	The voltage level at which the sub comments are referring to.

**Elements**			

**Name**	**Required**	**Data type**	**Description**

***Comment***	optional	String, see comment table for attributes	Comments can be used for collaboration and knowledge sharing amongst researchers and/or clinicians.

**Figure 6:**
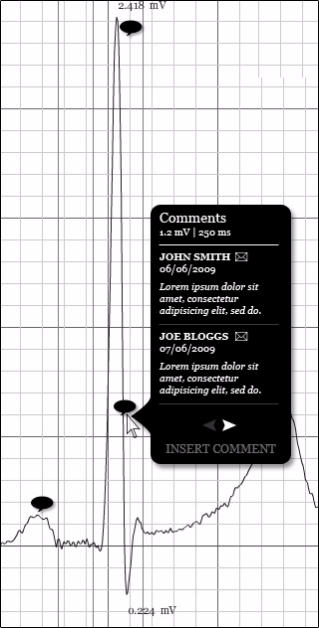
**Representing comments**. A mock-up of how the comments feature could be presented in a BSPM viewing system.

The ***comment ***element has four attributes. Three of these are required (***fullName*, *date*, *time***) and the other one is optional (***email***). The ***fullName ***attribute is required because the source of a comment is important and this is how most people assess the credibility of an observation. The ***date ***and ***time ***attributes are required, so that comments can be presented chronologically. The ***email ***attribute can either be used for identification or for correspondence. It is, however, optional since every user may not have or agree in submitting their email address. A full description of the comment element can be found in Table 9. Also, Figure 6 illustrates how this comments feature could be presented within a BSPM viewing application.

**Table 9:** Description of the comment element

Attributes			
**Name**	**Required**	**Data type**	**Description**

***fullName***	Required	Float	Storing the full name is useful for tracking discussions, e.g. Dr. John Smith.

***email***	Optional	Float	Email can be used for both identification and correspondence.

***date***	Required	Date YYYY-MM-DD	E.g. 12:50:00:000.

***time***	Required	Time:HH:MM:SS:SSS	E.g. 2009-08-28.

The ***limbLeads ***element is used to store any limb leads that were recorded to calculate the WCT. This element is optional since not all BSPM datasets retain limb lead data. Nevertheless, retaining limb lead data may prove useful for post processing. This element has one sub element called ***limbLead ***which can appear multiple times. The ***limbLead ***element has one required attribute called ***name***, which stores the name of the limb lead, i.e. I, II etc. Legacy limb leads can also be stored such as the VF, VR and VL leads given that legacy datasets and in particular the Kornreich-117 dataset [26] retains these limb leads. The ***limbLeads ***element and sub elements are described in Table 10.

**Table 10:** Description of the limbLeads element

Elements			
**Name**	**Required**	**Data type**	**Description**

***limbLead***	Required	String	Stores limb lead data. Multiple values are stored as CSV.

**Attributes**(for ***limblead ***element)			

**Name**	**Required**	**Data type**	**Description**

***name***	Required	aVF/aVR/aVL/I/II/II/VF/VR/VL	Stores the name of the limb lead.

The ***transformations ***element can be used to store any required equations required for further post processing. For example, it could be used to store equations for extracting the 12-lead ECG or the vectorcardiogram (VCG) from the BSPM dataset. Another example would be to store equations that could transform the BSPM into another BSPM dataset [27]. The ***transformations ***element has one optional sub element called ***transformation***, which may appear multiple times. A description of the ***transformations ***element can be seen in Table 11.

**Table 11:** Description of the transformations element

Elements			
**Name**	**Required**	**Data type**	**Description**

***transformation***	Required	See transformation table.	This element, for example can store equations for extracting the 12-lead ECG from the BSPM. It can also be used to transform one BSPM dataset into another.

The ***transformation ***element as described in Table 12 has one sub element called ***transformLead***, which can appear multiple times. It also contains one required attribute called ***name***, which stores the actual name of the transformation, for example, 12-lead ECG. The ***transformation ***element has another optional attribute called ***url***, which is used to retain a link to an XML file containing the actual calculations. By using this ***url ***attribute, BSPM files can share the same transformation equations. The ***transformLead ***element has one required attribute (***name***) and four optional attributes (***x, y, location, myocardialLocation***). These four attributes have been borrowed from the main ***lead ***element. They are optional here given that such information is not always relevant to every transformation. The ***name ***attribute is mandatory as it stores either the lead number or the lead name, i.e. ***aVF***. Finally, the ***transformLead ***element stores the actual equation for calculating a lead. A description of the ***transformLead ***element can be found in Table 13.

**Table 12:** Description of the transformation element

Attributes			
**Name**	**Required**	**Data type**	**Description**

***name***	Required	String	This attribute stores the name of the transformation dataset, i.e. 12-lead ECG, VCG or BSPM.

***url***	Optional	String	This attribute retains a link to an XML file containing the calculations.

**Elements**			

**Name**	**Required**	**Data type**	**Description**

***transformLead***	Required	See transformLead table.	*This stores an equation which when executed calculates a new lead.* *e.g. ([Lead52] + [Lead53])/2*

**Table 13:** Description of the transformLead element

Attributes			
**Name**	**Required**	**Data type**	**Description**

***name***	Required	String	This attribute stores the name of the lead, e.g. aVF.

***x***	Optional	Float	Stores the × axis for deriving the electrode position in relation to the thoracic diagram.

***y***	Optional	Float	Stores the y axis for deriving the electrode position in relation to the thoracic diagram.

***location***	Optional	A/P/LL/RL	Stores the general thoracic area of where the electrode was attached.

***myocardialRegion***	Optional	An/HP/TP/IP/I/L/Ap/RV/S	Refers to the corresponding myocardial region.

The rationale for storing equations in a BSPM format is that there are many custom electrode layouts. Therefore, each layout warrants its own unique set of equations for deriving, for example, the 12-lead ECG. Such calculations are also required as BSPM configurations do not usually contain the 12-lead ECG precordial electrode positions as a subset. Nevertheless, there is at least one BSPM lead configuration which has the six precordial leads as a subset of the BSPM leads [28], but most BSPM systems do not include all six precordial leads.

**Figure 7:**
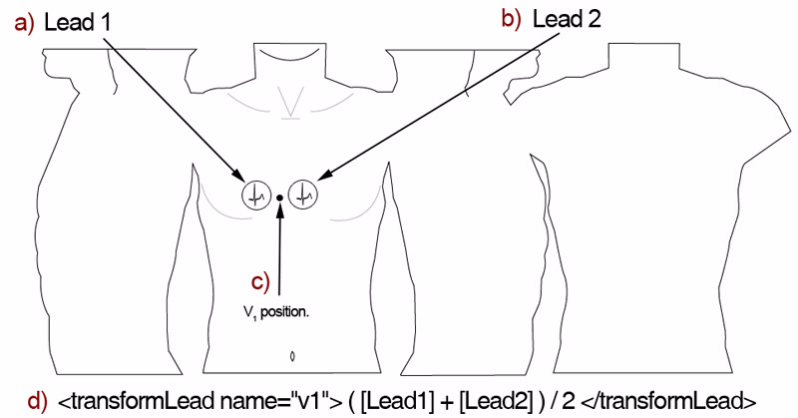
**transformLead element**. This diagram depicts how the transformLead element might be used to store a mean equation for calculating lead V1.

The main challenge when creating the ***transformLead ***element was defining a method for storing equations. One can define an equation using normal mathematical syntax; however, a simple method has been set in place for referring to individual BSPM leads. This method can be seen in Figure 7. This diagram illustrates a simple two lead BSPM that has been stored using this XML format. Suppose that these two leads where recorded from the precordial region and that an imaginary V1 lead from a 12-lead ECG could be positioned midway between the two BSPM leads. If this was the case, then a pseudo V1 lead could be calculated by storing an equation within the ***transformLead ***element. This is illustrated in section D of Figure 7. This is a simple mean equation that adds each value from leads one and two and averages them by dividing them by two. This equation could be represented within the ***transformLead ***element as "([Lead1] + [Lead2])/2". The square brackets encompassing the word 'Lead' followed by the actual BSPM lead number is the syntactical method used within this format to reference a BSPM lead in an equation.

The ***diagram ***element is a required sub element of the ***header ***tag. It is required as it stores an unrolled torso schematic that is used as a reference diagram in a 2D coordinate system. This 2D coordinate system is used for storing electrode positions relative to the torso. This format must store electrode positions, in order to support different BSPM electrode layouts. Knowledge of electrode positions is also useful for clinical reference and visualizing the BSPM, i.e. contour plotting. Interestingly, like the BSPM, the number of electrodes used in certain instances to record an electroencephalogram (EEG) is not as standardised. These electrodes can also be positioned at custom cranial landmarks. Therefore, since the EEG and the BSPM are not as standardised as the 12-lead ECG, some EEG formats such as the Extensible Biosignal Format (EBS) store the actual electrode positions. The EBS format was created in 1993 by Marcus Kuhn and the specification is freely available online [29]. The EBS format stores the electrode positions within the header of the file using a Computer Graphics Metafile (CGM). These EEG formatting concepts provided inspiration in the development of the proposed BSPM format.

The ***diagram ***element has two optional attributes called ***url ***and ***waveScale***. The ***url ***attribute is an abbreviation for Uniform Resource Locator (URL). This attribute stores a path or a web address to a torso diagram, which can reside either on the internet or on a local network. The ***waveScale ***attribute stores a float value between zero and one. This value is used to proportionally scale BSPM leads (scalar traces) to fit comfortably in their associated electrode positions, in relation to the size of the torso diagram. For example, the value 0.1 would scale the waveforms to 10 percent of their actual size. Although this attribute is optional, if it does not exist then the value defaults to 0.04, which scales all waveforms to 4% of their actual size. This default value has been chosen, as it proportionally scales waveforms in relation to torso diagrams that are approximately the size of an A4 piece of paper. This is a standard paper size within the UK.

The advantage of implementing a format in XML as opposed to binary is its accessibility to a suite of related XML technologies, i.e. Scalable Vector Graphics (SVG). SVG is a W3C recommendation and an XML language for describing images [30]. The ***diagram ***element stores an unrolled 2D torso schematic using SVG. This element is similar to the idea suggested in another study [31], which is to integrate a photograph of the subject into the format, in order to retain electrode locations.

**Figure 8:**
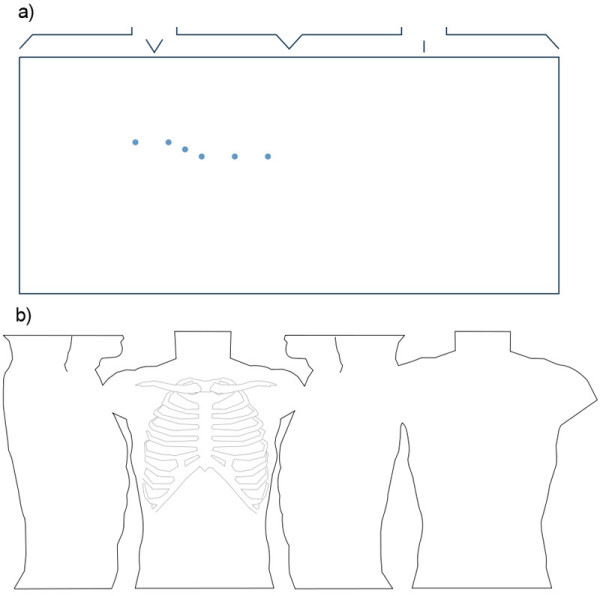
**SVG torso diagrams**. a) A simple one kilobyte SVG torso diagram. b) A more complex nine kilobyte torso diagram, which includes drawings of the intercostal spaces.

Raster images bulk up the size of the file and a photograph does not give direct programmatic access. SVG images scale better and are smaller in terms of file size when compared to rasterised equivalents. An example of a simple SVG torso diagram is depicted in Figure 8a[16]. This particular SVG diagram is just one kilobyte in size and 400 bytes when compressed (ZIP), whereas the rasterised (JPEG) equivalent is 50 kilobytes. As of yet, there has been no standard torso schematic proposed for displaying BSPMs. As previously mentioned, currently researchers draw their own custom torso diagrams, which is why the proposed BSPM format allows for the integration of custom torso diagrams using SVG. Although Figure 8a is a basic torso diagram, SVG has the ability to describe complex torsos such as the one depicted in Figure 8b. Unfortunately, intricate diagrams usually mean larger file sizes.

**Figure 9:**
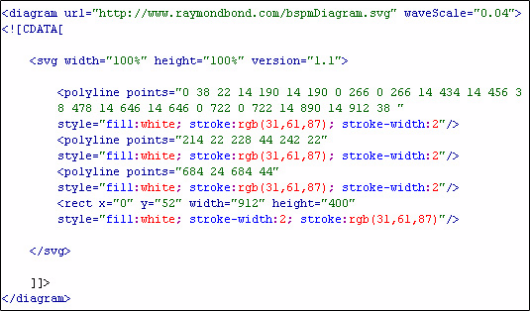
**Embedded SVG diagram**. This is an excerpt of the XML-BSPM format depicting the storage of the SVG diagram.

There are three options when integrating an SVG diagram into the BSPM format. Method one is to embed SVG markup directly into the ***diagram ***element. The disadvantage is that, a large embedded SVG diagram will increase the size of the document and make it less readable. The advantage of this method is that, it excludes the need for network or internet access for loading an external SVG file. Method two is to reference a URL using the ***url ***attribute, which stores a path to an SVG diagram. This method does reduce the size of the file; however, it relies on external access to an SVG file. If the external file no longer exists, then the BSPM file becomes redundant, since a diagram is required to establish the electrode positions. This method is also disadvantageous as multi-file management can be a problem [8]. Finally, method three combines both the previous methods and has been adopted in the current work. This approach stores a URL linking to a complex SVG torso diagram, i.e. a torso outline with anatomical structures such as a rib cage, whilst embedding a simplistic version of that diagram. In effect, when a computer program cannot load the external complex SVG file, it can alternatively load the simplistic embedded version. Therefore, method three keeps the file size at a minimum whilst retaining the capability of loading a sophisticated torso diagram. Method three is also advantageous because it can always retain its electrode positions. Even if the external SVG file is lost, it utilises the embedded version in its place. An example of method three can be viewed in Figure 9. The data type for the ***diagram ***element is Character Data (CDATA). CDATA allows the element to store XML markup without it being processed. This means that web browsers will display the BSPM format in the normal XML tree structure. Otherwise, without CDATA as the data type, some web browsers would disrupt the tree structure by rendering the SVG diagram on top of the XML. Also, the absence of the CDATA data type would complicate the format and demand namespace management. A description of the diagram element can be found in Table 14.

**Table 14:** Description of the diagram element

Attributes			
**Name**	**Required**	**Data type**	**Description**

***url***	Optional	String	This attribute can store an external link to an SVG diagram, e.g.: http://www.raymondbond.com/bspmDiagram.svg

***waveScale***	Optional	Float, DEFAULT: 0.04	This value is used to proportionally scale the waveforms in relation to the size of the torso diagram, e.g.: "0.04" scales the leads to 4% of their actual size.

The ***leads ***element is a sub element of the root element, ***bspm***. This element acts as a wrapper tag that accumulates all the subordinate BSPM leads. Hence, the ***leads ***element has one sub element called ***lead***, which can appear multiple times. As its name suggests, each ***lead ***element stores a unipolar ECG lead. Other ECG formats store lead data using a different name, i.e. aECG [13] uses the name 'digits' and the ecgML [7] format uses the name 'channel'.

**Figure 10:**
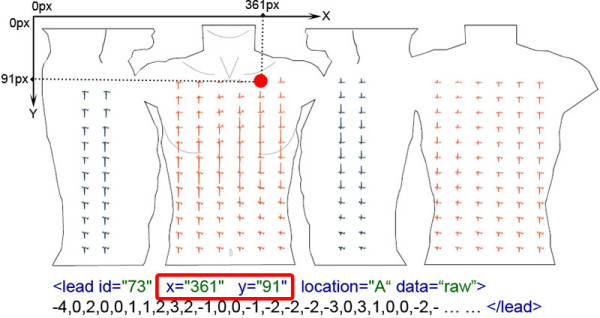
**Storing electrode positions**. This diagram illustrates a 2D coordinate system of how the attributes x and y are used to derive an electrode position.

The ***lead ***element as described in Table 15 has six attributes, three of which are optional and three that are required. The ***id ***attribute is a requirement, since it simply stores the lead number. For example, to store the Lux-192 BSPM dataset, there would have to be 192 ***lead ***elements within the ***leads ***element. The ***x ***and ***y ***attributes are also required. These attributes store numerical pixel values which are, in effect, 2D coordinate values. These coordinate values are used to derive electrode positions, which is performed with reference to the SVG torso schematic that is stored within the ***diagram ***element. This 2D coordinate system for storing electrode positions is demonstrated in Figure 10.

**Table 15:** Description of the lead element

Attributes			
**Name**	**Required**	**Data type**	**Description**

***Id***	Required	Integer	This integer is used to uniquely identify and number each lead.

***X***	Required	Float	Stores the × axis for deriving the electrode position in relation to the thoracic diagram.

***Y***	Required	Float	Stores the y axis for deriving the electrode position in relation to the thoracic diagram.

***Location***	Optional	A/P/LL/RL	Stores the general thoracic area of where the electrode was attached.

***myocardialRegion***	Optional	An/HP/TP/IP/I/L/Ap/RV/S	Refers to the corresponding myocardial region.

***Data***	Optional	raw/calc DEFAULT: 'raw'	Defines whether the lead stores raw data or a calculation.

**Figure 11:**
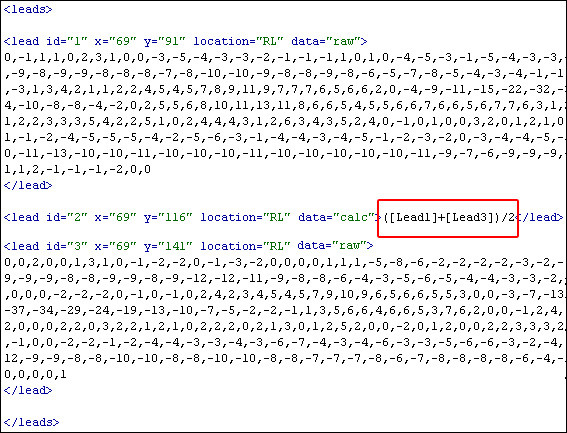
**Calculated leads**. This is an excerpt of the leads element where the type attribute has been used to distinguish between calculated leads and raw leads.

The ***location ***attribute is optional and is used to define which portion of the torso, the electrode was positioned. This attribute can have four possible abbreviated values, i.e. ***LL ***(Left Lateral), ***RL ***(Right Lateral), ***A ***(Anterior) and ***P ***(Posterior). Defining the general location of each electrode, allows a computer program to best visualise and distinguish between leads, for example, anterior leads could be rendered a different colour from the posterior leads. The ***myocardialRegion ***attribute is used to define the corresponding region of the myocardium each lead refers to. The nine possible values are abbreviated, i.e. Ap stands for apical. Using this attribute, a computer program can assess ST elevation in the appropriate leads for diagnosing acute myocardial infarction. Finally, the ***data ***attribute is also optional and is used to define whether the content of the ***lead ***element contains raw CSV values or an equation, that when executed calculates lead data. Hence, the data attribute can only have one of two values, i.e. ***raw ***or ***calc ***(calculation). The ***raw ***value indicates that the ***lead ***element contains raw ECG values and the value ***calc ***indicates that the lead element contains an equation for deriving the lead. This is illustrated in Figure 11. Although, the ***type ***attribute is optional, if no value exists, then the value defaults to ***raw***. Moreover, the ***type ***attribute should only be used if the ***type ***attribute in the ***bspm ***element has the value ***AVERAGED-BEATS-BSPM-TRANSFORM ***or the value ***CONTINUOUS-BEATS-BSPM-TRANSFORM***.

## Results

### Evaluation of the format

**Figure 12:**
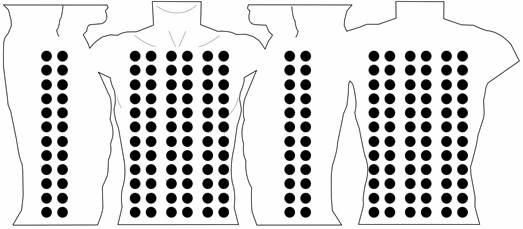
**Lux-192 BSPM**. Lux-192 electrode layout consisting of 192 electrodes equally spaced throughout a custom torso diagram.

This format has the potential to support the storage of all available BSPM datasets. It has been tested to store two BSPM datasets that have been widely reported in the literature. The Lux-192 dataset [32] consists of a 12 × 16 array of 192 leads (electrodes) as illustrated in Figure 12. Since all leads in a BSPM are unipolar, the terms lead and electrode may be used interchangeably. The rows in the Lux-192 layout are equally spaced between the suprasternal notch and the umbilicus. The columns are also equidistant around the whole thorax. Each lead contains a single averaged beat (approximately 600 sample values) sampled at 1000 Hz. That equates to, approximately, 100, 000 data values in one file. This dataset has been stored using the proposed BSPM format. The file size was 293 kilobytes and was later compressed to 76 kilobytes using the ZIP compression algorithm. Also, the GZIP algorithm was used and was able to compress the file to just 68 kilobytes. As previously discussed, it can be said, that file size is not as important as it once was. However, telemedical systems may rely on small mobile devices, which rely on constrained processing environments and smart cards for storing data. In this context file size becomes very important.

**Figure 13:**
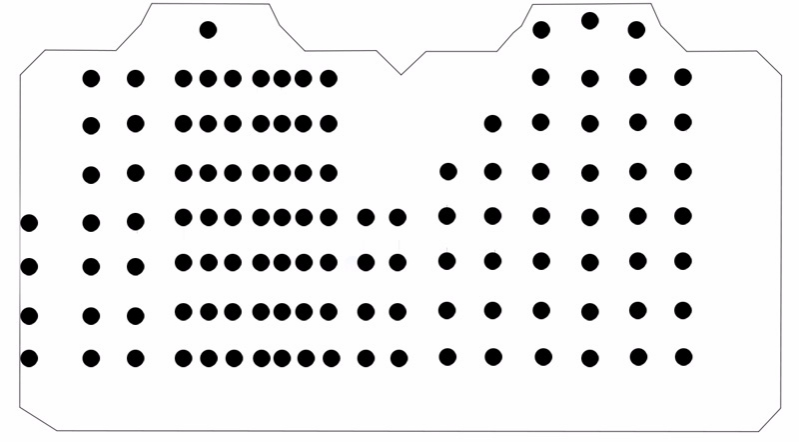
**Kornreich-117 BSPM**. Kornreich-117 electrode layout consisting of 117 electrodes placed throughout a custom torso diagram.

Another BSPM dataset has been stored using this BSPM format, namely the Kornreich-117 lead set [26], which can be seen in Figure 13. The placement of electrodes in this layout is not as symmetrical as the Lux-192 lead set, i.e. the spacing between the columns in the Kornreich-117 is not equidistant. This BSPM dataset consists of 117 averaged beats (approximately 300 sample values) and was sampled at 500 Hz. That equates to approximately 35,000 data values within one file. Unlike the Lux-192 dataset, the Kornreich-117 dataset retains three limb leads (VL, VR and VF) that were used to calculate the WCT. This means, a further 900 (approximately) data values have been stored within the ***limbLeads ***element. One Kornreich-117 dataset was stored at 257 kilobytes. This file was later reduced to 110 kilobytes using the ZIP compression algorithm. Alternatively, the GZIP compression algorithm reduced the file to 111 kilobytes. These results have been summarised in Table 16.

**Table 16:** A comparison of BSPM file sizes when the file is stored as XML or compressed using either the ZIP or GZIP compression algorithms

BSPM dataset	Raw XML	ZIP compression	GZIP compression
**Lux-192 BSPM**	293 kilobytes	76 kilobytes	68 kilobytes

**Kornreich-117 BSPM**	257 kilobytes	110 kilobytes	111 kilobytes

To truly evaluate this format and in particular, its file size, it would seem appropriate to compare these files with other BSPM files that have been formatted by a different standard. This is complicated by the fact that no other BSPM format exists. It would also be difficult to compare BSPM files with 12-lead ECG files that have been formatted with, for example SCP-ECG, since the storage of 8 leads and 200 leads are not comparable. Some 12-lead ECG files formatted using the aECG format can be as large as 500 kilobytes, which is a lot larger than the BSPM files that have just been evaluated within this study.

### Evaluation of the transformation feature

**Figure 14:**
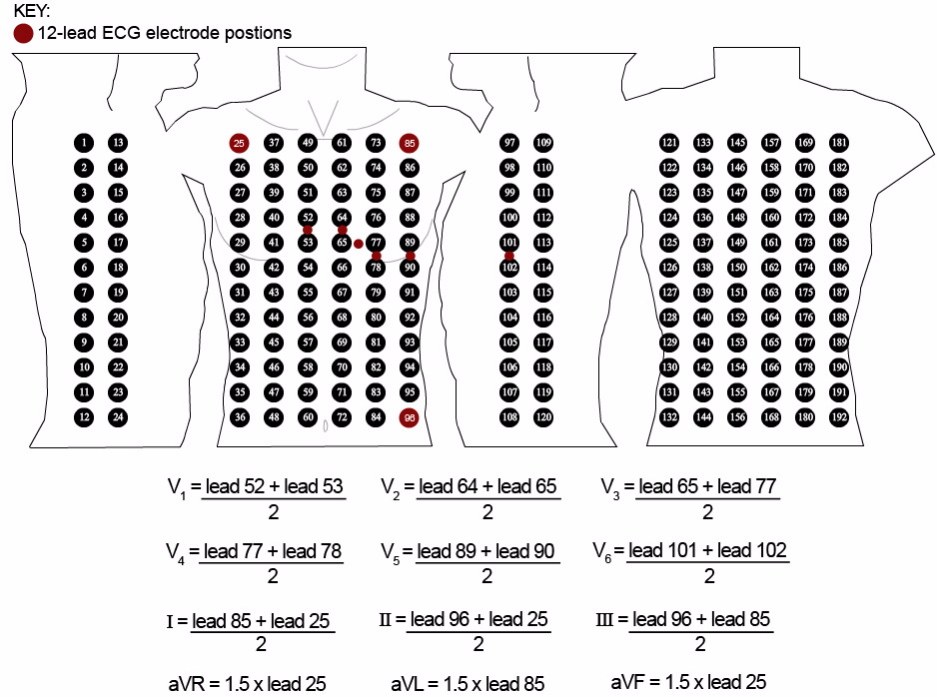
**Extracting the 12-lead**. The 12-lead ECG in relation to the Lux-192 BSPM and the basic equations used for extracting the 12-lead ECG from the BSPM.

Within the methods section of this paper, a technique was described for storing equations within the ***transformations ***element of the BSPM format. This method has been tested to define equations for extracting both a 12-lead ECG and a VCG from a BSPM. A Lux-192 BSPM [32] was stored using this XML format. Figure 14 illustrates a study carried out by Drew *et al*. [33], which demonstrates the 12-lead ECG electrode positions in relation to the Lux-192 electrode positions. This diagram allowed us to determine the basic calculations required to extract a 12-lead ECG from a Lux-192 BSPM. The red circles indicate the 12-lead ECG electrode positions. When this particular dataset was recorded, the WCT was determined from standard (distal) limb leads. Unfortunately, this original limb lead data was not kept. As a result, the Mason and Likar (proximal) limb electrode positions have been used and yield the exercise variant of the 12-lead ECG. Lead number 25 represents the right arm electrode, lead 85 represents the left arm electrode and lead 96 represents the left foot electrode. However, if the raw limb leads where kept and stored within the header of the format, such limb leads could have been used within these equations. This can be performed using the keyword "limbLead" concatenated with the name of the lead, i.e. "[limbLeadaVF]". As acknowledged, the Lux-192 dataset did not retain the original limb lead data, but can be obtained from the Mason and Likar torso leads. Moreover, the six precordial leads do not directly match any of the 192 leads. As a result, we define basic interpolative calculations. For example, lead V1 would be positioned midway between leads 52 and 53 of the Lux-192 electrode layout. Therefore, a simple mean calculation as defined in Figure 14 can be used to calculate this lead.

**Figure 15:**
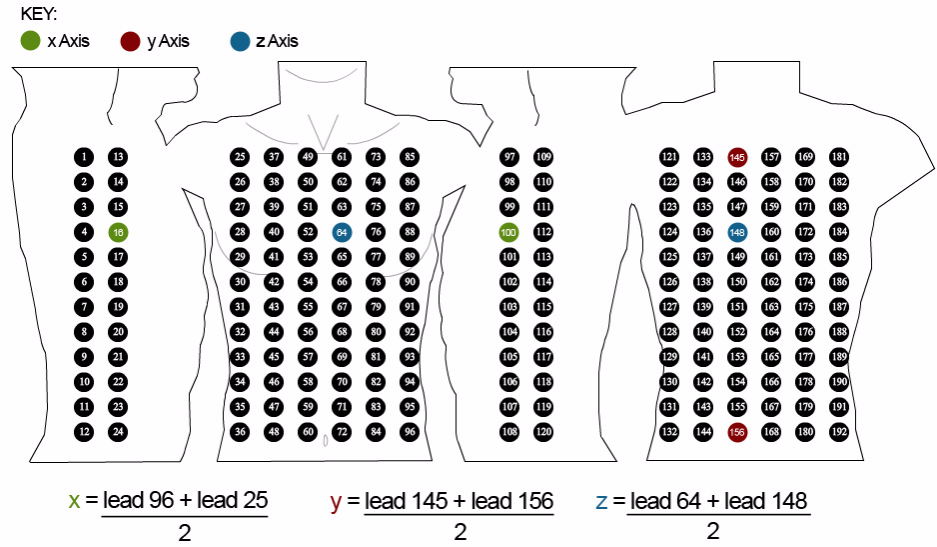
**Extracting the VCG**. This diagram highlights the leads from the Lux-192 BSPM and the equations used to calculate the VCG.

**Figure 16:**
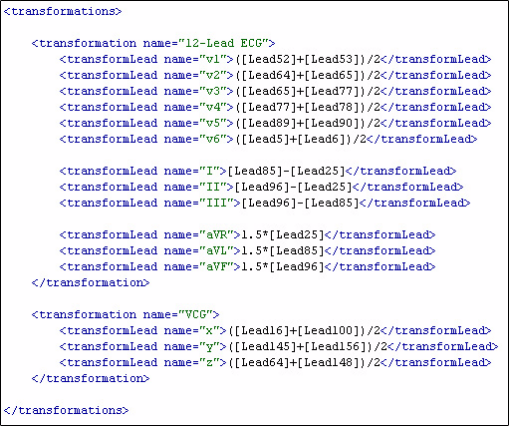
**12-lead ECG and VCG equations**. This diagram contains the 12-lead ECG and the VCG equations as defined within the XML-BSPM format.

As well as defining equations for deriving the 12-lead ECG, equations can also be defined to derive the VCG based on Frank lead system [34]. Figure 15 indicates the Frank based VCG electrode positions in relation to the Lux-192 layout [35]. Fortunately, all of the VCG recording sites are a subset of the BSPM electrodes. The green circles represent the × axis, red circles represent the y axis and the blue circles represent the z axis. Lead number 16 can be used as lead I and lead 100 can be used as lead A. The potential difference between leads I and A creates the × axis of the VCG. Lead 145 can be used as lead H and lead 156 can be used as lead F. The potential difference between leads H and F make up the y axis. Finally, the z axis is the potential difference between lead 64 and lead 148, better known as leads E and M in the context of VCGs. Figure 15 illustrates the calculations used to derive the x, y and z axes of the VCG. The 12-lead ECG and VCG equations can be seen in Figure 16. This figure shows how the equations look within the actual BSPM format.

## Discussion

### Accompanying tools

**Figure 17:**
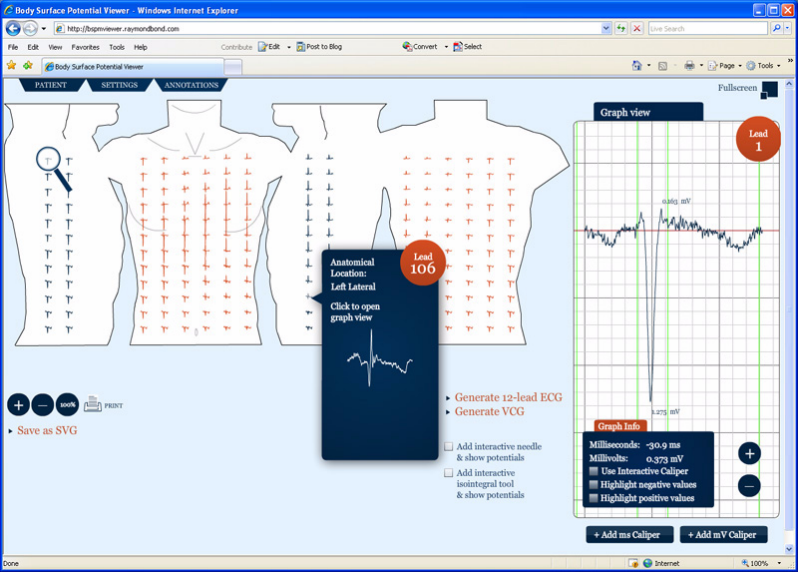
**BSPM viewer**. A web based BSPM viewer for parsing, processing and visualising XML-BSPM files.

Since visual representation demonstrates a format, a web based BSPM viewer is currently underdevelopment for staging the capabilities of this BSPM format. The current version of the system can be found online [36]. This Rich Internet Application (RIA) parses the format, renders the SVG torso diagram, draws the BSPM leads and provides interactive tools to allow clinicians to intuitively explore the BSPM. These tools include callipers, an isopotential and an isointegral tool. This BSPM viewer was created using the Adobe Flash technology [37]. This application is geared towards both the clinician and the engineer, whereas traditional BSPM tools were designed only for the engineer, i.e. Map3D [38]. A screenshot of the BSPM viewer can be seen in Figure 17.

### Future work

The format proposed in this paper has been developed to the level where it is useable. However, a number of additional tests are foreseen to prove the full utility of the format. These tests include the ability to securely store long term/continuous data as opposed to averaged beats and the ability to support data recorded from limited lead sets. Further work will also include the completion of the web based BSPM viewer, a format validation service, a BSPM to SVG converter and possibly an online warehouse, where users can share and download BSPMs. In summary, this research provides initial ground work for creating a complete BSPM management system.

## Conclusions

The work presented in this paper documents one of the first attempts to establish a storage format that supports data recorded from any BSPM recording configuration. The primary goal has been to promote the storage and sharing of data that traditionally has not been widely accessible. Although the format has been conceived, bearing in mind the requirements of storing data recorded from a large number of leads, it can in fact be used to support data from any ECG recording. That said, this proposed format should not be seen as a competitor to well established formats such as SCP-ECG, DICOM and aECG. The lessons learned in this work and reported in this paper can be used for the further development of existing ECG formats and standards; which in the future may be enhanced to support BSPM leads.

## Competing interests

The authors declare that they have no competing interests.

## Authors' contributions

RRB carried out the literature review, designed the XML schema for storing BSPMs and drafted this manuscript. DDF, CDN and GM participated in the coordination and design of this study and also helped draft this manuscript. All authors read and approved the final paper.

## Pre-publication history

The pre-publication history for this paper can be accessed here:

http://www.biomedcentral.com/1472-6947/10/28/prepub
